# Supplementary material for: Multi-heme cytochrome-mediated extracellular electron transfer by the anaerobic methanotroph ‘Candidatus Methanoperedens nitroreducens’
Source: Nat Commun. 2023 Sep 30;14:6118. doi: 10.1038/s41467-023-41847-w (PMC10542353; doi:10.1038/s41467-023-41847-w)
Supplement: Supplementary file 1 — Supplementary Information [file 41467_2023_41847_MOESM1_ESM.pdf]

Supplementary Information for

Multi-heme cytochrome-mediated extracellular electron transfer by the anaerobic methanotroph '*Candidatus* Methanoperedens nitroreducens'

Xueqin Zhang[1], Georgina H. Joyce[2], Andy O. Leu[2], Jing Zhao[1,3], Hesamoddin Rabiee[1,4,5], Bernardino Virdis[1], Gene W. Tyson[2], Zhiguo Yuan[1,6], Simon J. McIlroy[2], Shihu Hu[1*]

[1]Australian Centre for Water and Environmental Biotechnology (ACWEB), Faculty of Engineering, Architecture and Information Technology, University of Queensland, Brisbane, Australia

[2]Centre for Microbiome Research, School of Biomedical Sciences, Queensland University of Technology (QUT), Translational Research Institute, Woolloongabba, Australia

[3]Ecological Engineering of Mine Wastes, Sustainable Minerals Institute, The University of Queensland, Brisbane, Queensland, Australia

[4]School of Chemical Engineering, The University of Queensland, Brisbane, Queensland, Australia

[5]Centre for Future Materials, University of Southern Queensland, Springfield, Queensland, Australia

[6]School of Energy and Environment, City University of Hong Kong, Hong Kong SAR, China

*Corresponding author. Email: s.hu@uq.edu.au

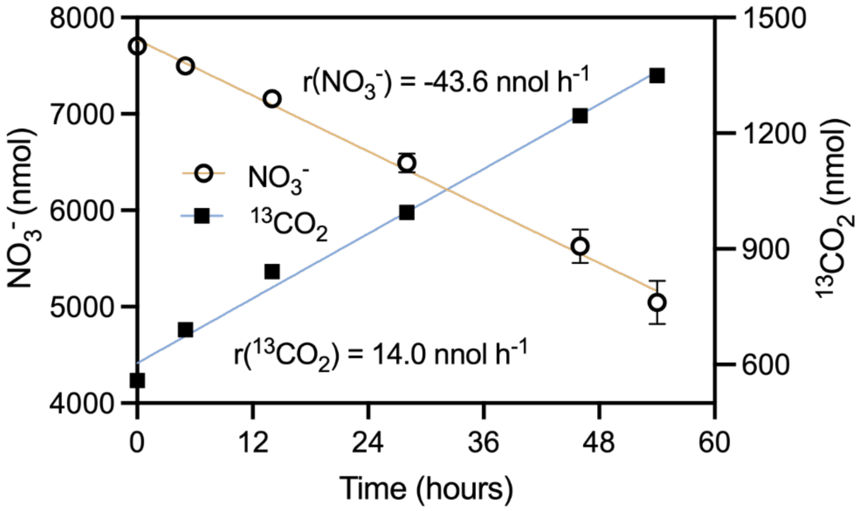

Supplementary Fig. 1 Performance of nitrate-dependent AOM by '*Ca.* M. nitroreducens' enrichment culture. NO_3^- reduction and $^{13}CO_2$ production in the control treatment incubation with nitrate (5 mM) as the electron acceptor. Source data are provided as a Source Data file.

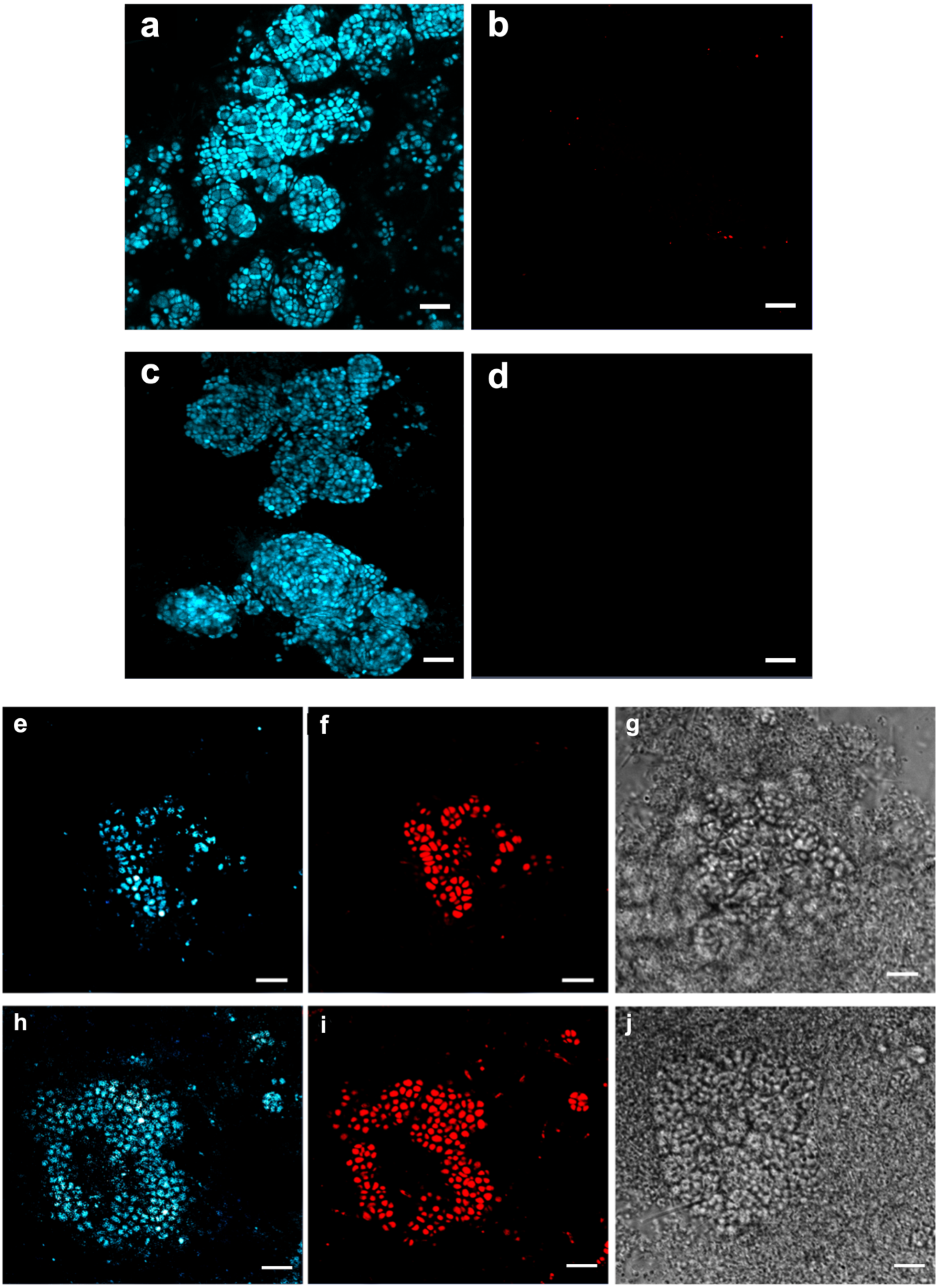

Supplementary Fig. 2 Representative fluorescence micrographs of '*Ca.* M. nitroreducens' cells represented by autofluorescence and iron-reducing cells represented by Fe^{2+}-selective chemosensory fluorescence. **(a, b)** Fluorescence images from the control fluorescent assay, in which '*Ca.* M.

nitroreducens' enrichment culture was fed with 5 mM Fe^{3+} and the mixture was immediately stained with 25 μM Fe^{2+}-specific probe. (**c**, **d)** Fluorescence images from a control fluorescent assay where the microcosm collected at the end of iron-reducing incubation was not stained with Fe^{2+}-specific probe. (**e-j**) cells processed with both iron-reducing incubation and staining of 25 μM Fe^{2+}-specific probe. (**a, c, e, h**) Autofluorescence of '*Ca.* M. nitroreducens' cells exhibited by the methanogenic cofactor F_{420}. (**b, d, f, i**) fluorescence images of the iron-reducing cells stained by FeRhoNox. (**g, j**) Phase-contrast micrograph at the corresponding fields of view. The experiment was repeated independently three times with similar results.

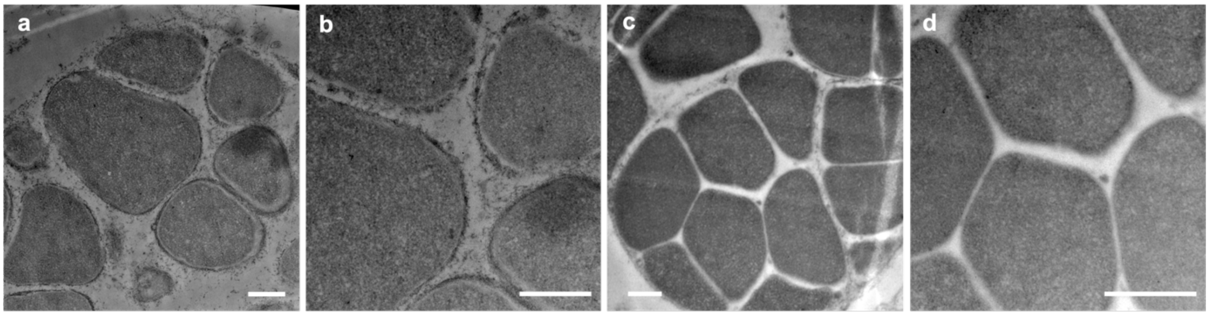

Supplementary Fig. 3 TEM images of '*Ca*. M. nitroreducens' cells stained with cytochrome-reactive DAB-H_2O_2. The experiment was repeated independently three times with similar results. (**a** and **b**) '*Ca*. M. nitroreducens' cells for inoculum of iron reduction batch (nitrate-adapted cells) with positive DAB staining in the presence of H_2O_2 and (**c** and **d**) negative DAB staining in the absence of H_2O_2.

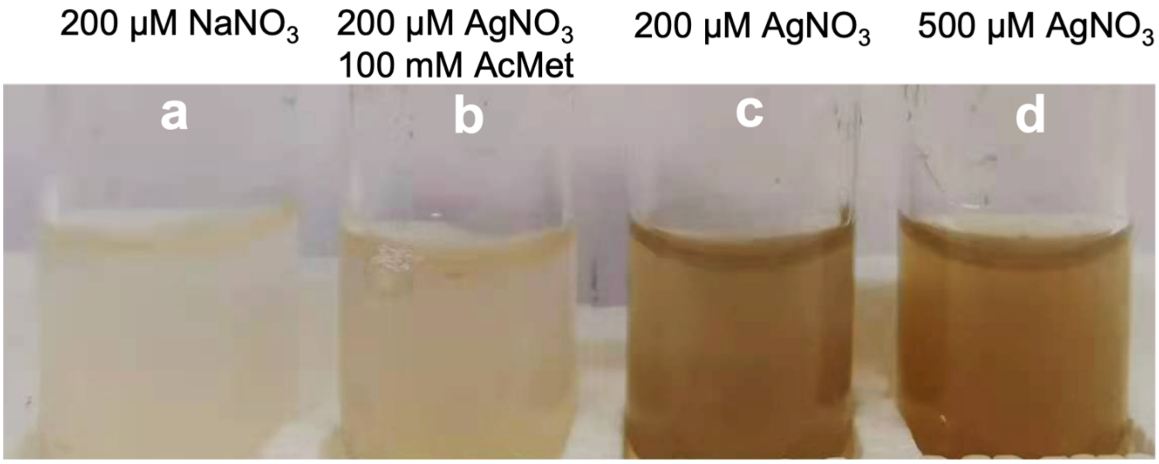

Supplementary Fig. 4 Photograph of visual changes in colour representing the formation of AgNPs from the reduction of Ag (I) by the '*Ca*. M. nitroreducens' enrichment culture incubations.

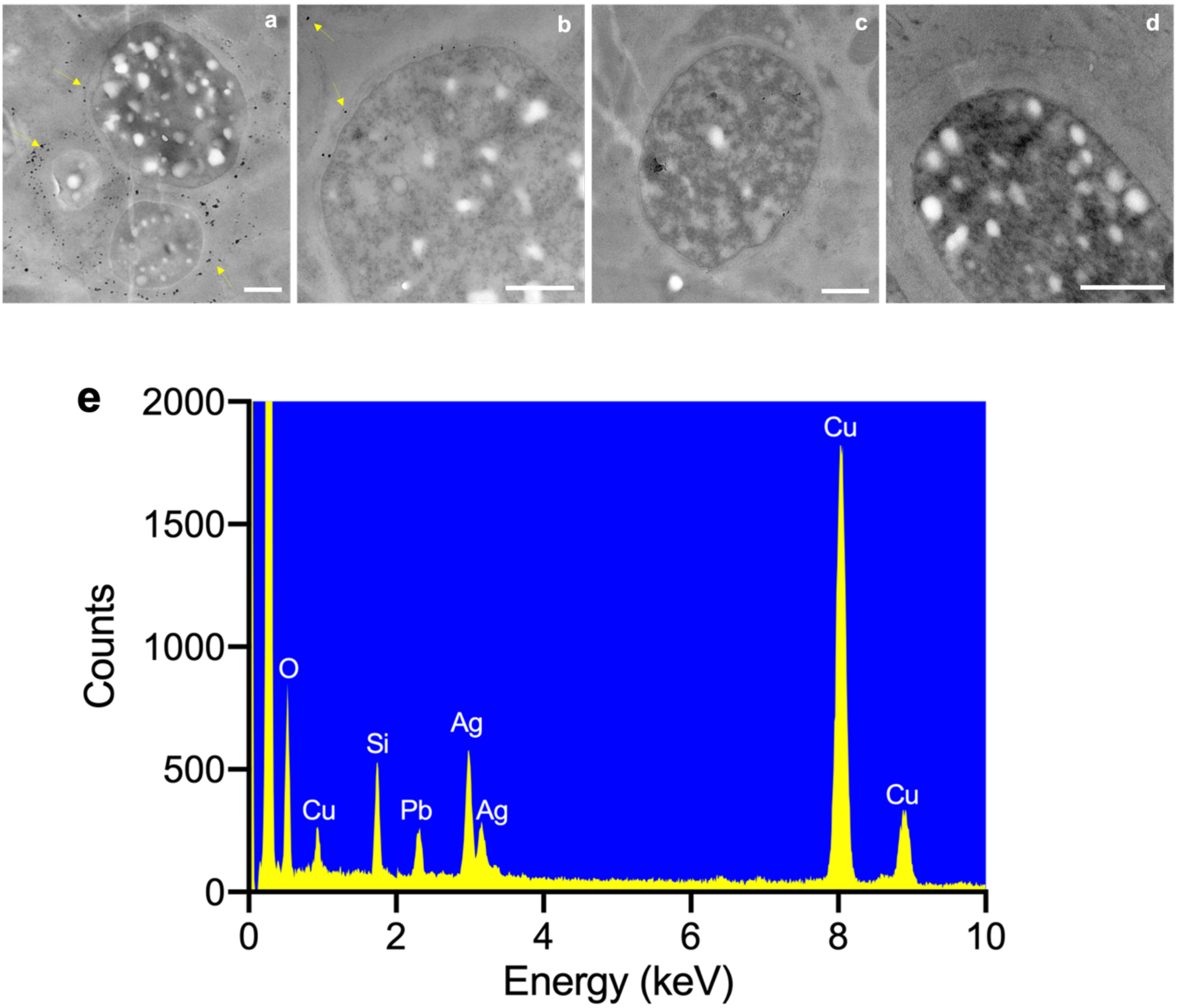

Supplementary Fig. 5 TEM visualization of Ag(I) reduction and deposition as Ag(0), and characteristic energy-dispersive spectroscopy (EDS) of deposited Ag(0) in the Ag (I)-fed batch incubations. Six independent experiments were conducted with similar results. (**a**, **b**) Ag(0) deposition (indicated by arrows) on the outer membrane of '*Ca.* M. nitroreducens' cells. (**c**, **d**) Ag(0) deposition was inhibited by AcMet. Scale bars, 500 nm. (**e**) A representative EDS spectrum of extracellular precipitates from sectioned samples.

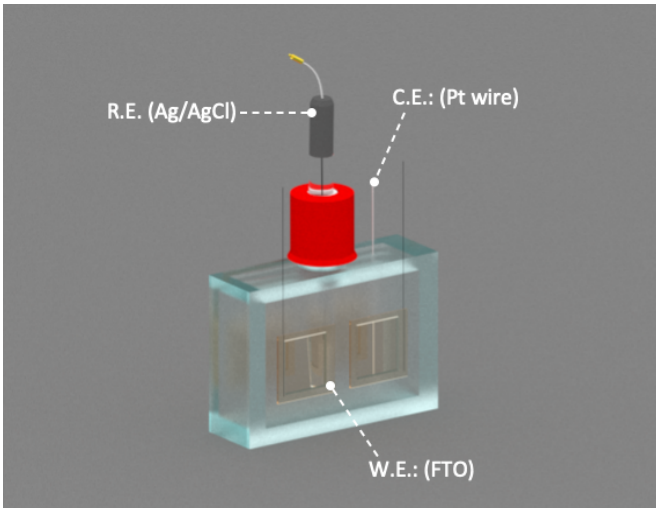

Supplementary Fig. 6 Schematic illustration of the electrochemical cell used for whole-cell electrochemical characterization.

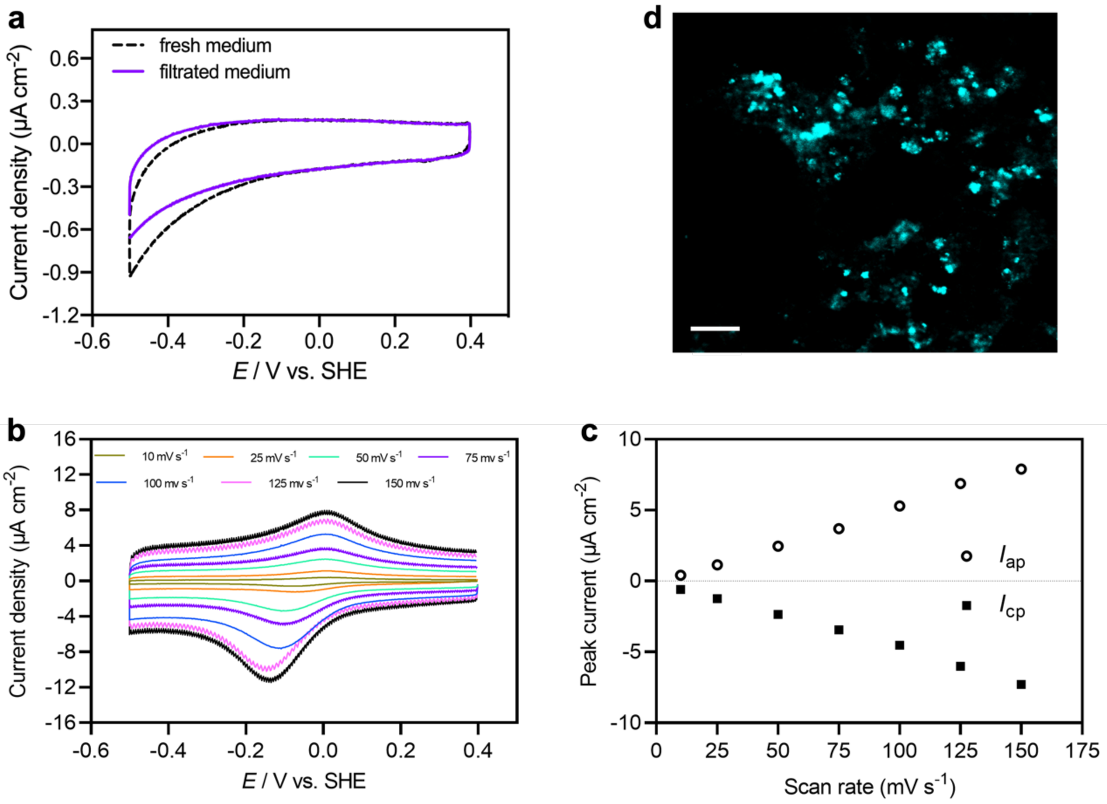

Supplementary Fig. 7 Electrochemical characterization and fluorescence micrograph showing the contact-based EET pathway of '*Ca.* M. nitroreducens' for electrode reduction. (**a**) Whole-cell CVs of fresh medium and of the cell-filtrated medium of '*Ca.* M. nitroreducens' enrichment culture that had been electrochemically cultivated at +0.4 V *vs.* SHE for 24 h. (**b, c**) Whole-cell CVs of '*Ca*. M. nitroreducens' enrichment culture at different scan rate. (**b**) CV curves at different scan rates. CVs were conducted with '*Ca.* M. nitroreducens' enrichment culture that was electrochemically (poised at + 0.4 V vs SHE) cultured for 24 h. (**c**) Plot of peak current as a function of the scan rate. (**d**) A fluorescence microscopy image of '*Ca*. M. nitroreducens' cells (represented by autofluorescence of methanogenic cofactor F_{420}) attached on the FTO electrode that was poised at 0.4 V vs SHE. Scale bar: 20 μm. Source data are provided as a Source Data file.

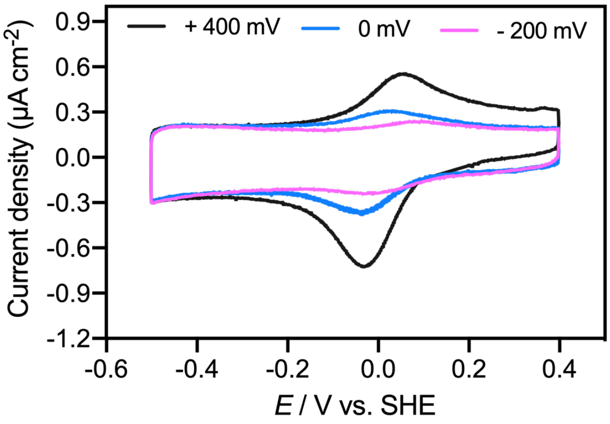

Supplementary Fig. 8 Whole-cell CVs of the '*Ca.* M. nitroreducens' enrichment culture that had been electrochemically incubated at different potentials (400 mV, 0 mV and -200 mV vs SHE) for 24 h prior to the measurements. Source data are provided as a Source Data file.

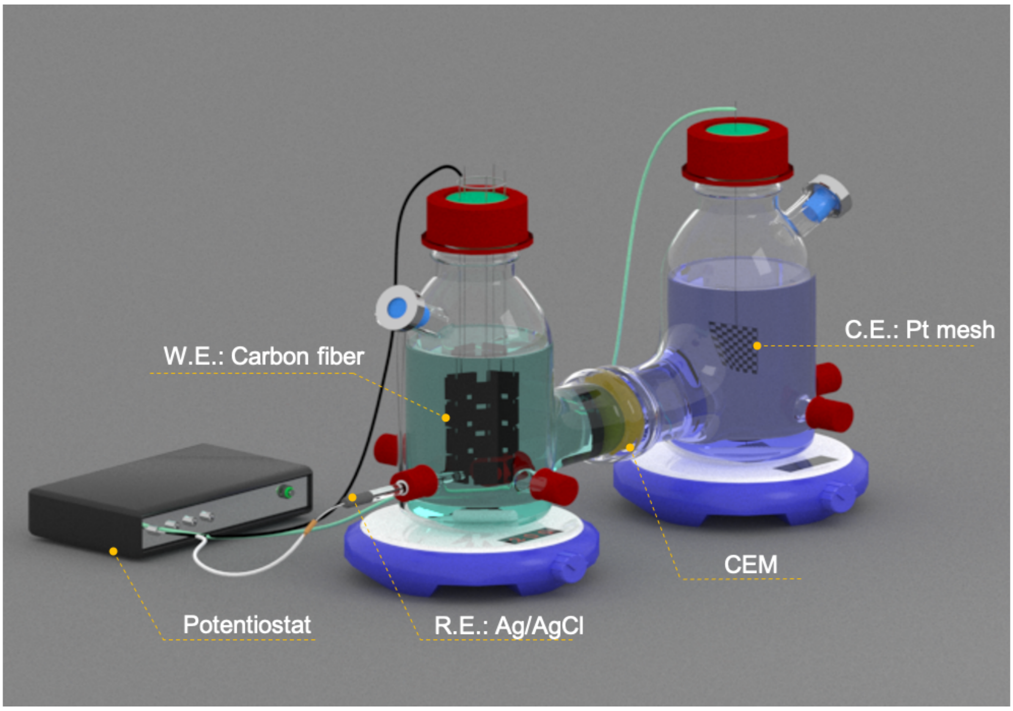

Supplementary Fig. 9 Schematic representation of the BES with multiple working electrodes

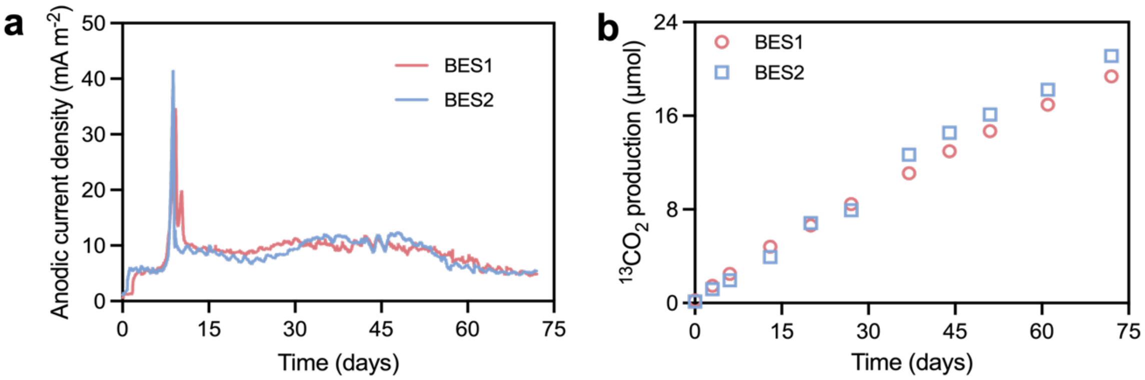

Supplementary Fig. 10 Performance of electrode-dependent AOM. (**a**) The anodic current generation in the replicate BESs (BES1 and BES2) operated for 72 days. (**b**) The oxidation of $^{13}CH_4$ to $^{13}CO_2$ in the BESs. Source data are provided as a Source Data file.

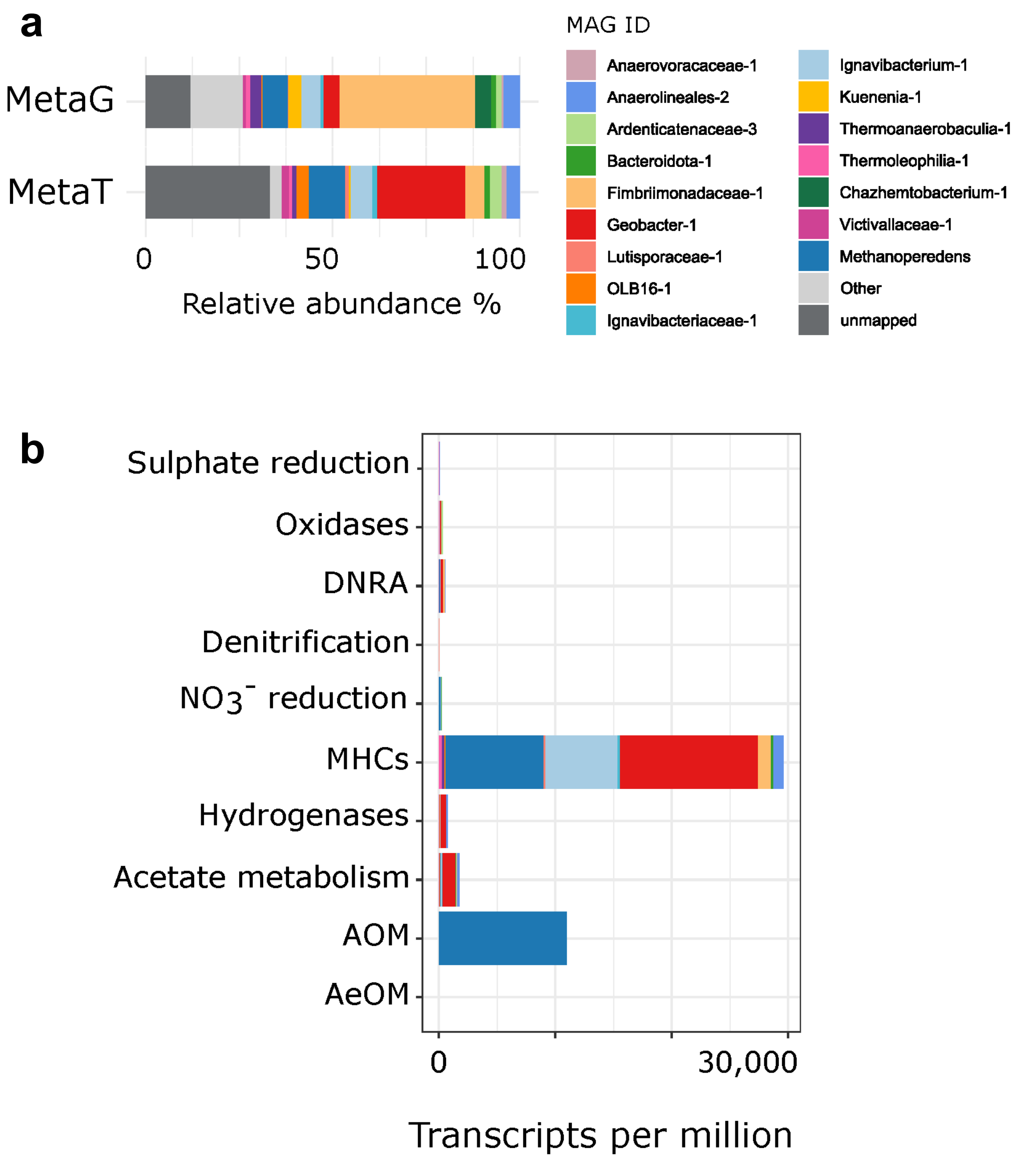

Supplementary Fig. 11 Metatranscriptomic profile of electrode-dependent AOM. (a) Microbial community metagenomic and metatrascriptomic profiles. (b) Community gene expression for selected metabolic pathways (Supplementary Table S4). Charts display the average of three biological replicates. 'Acetate metabolism' includes genes, *pta*, *acs*, *ack*, *acdA*. 'Sulphate reduction' includes dissimilatory sulphate reduction genes, *sat* and *aprA*. 'Oxidases' includes cytochrome c oxidase genes, *ccoN*, *ccoO*, *ccoP*, *coxA*, *coxB*. 'DNRA' is the abbreviation for 'Dissimilatory nitrate reduction to ammonium'. 'AeOM' is for 'Aerobic methane oxidation' includes *mmo* monooxygenase genes. 'AOM' is for 'Anaerobic oxidation of methane' and includes *mcr* reverse methanogenesis genes. MHCs were predicted by identifying proteins with more than three CXXCH amino acid motifs. 'Other' includes MAGs at less than 1% relative abundance in both the metagenome and metatranscriptome. Metagenome and metatranscriptome were conducted with electrode biofilm formed after 72 days operation of the bioelectrochemical reactor.

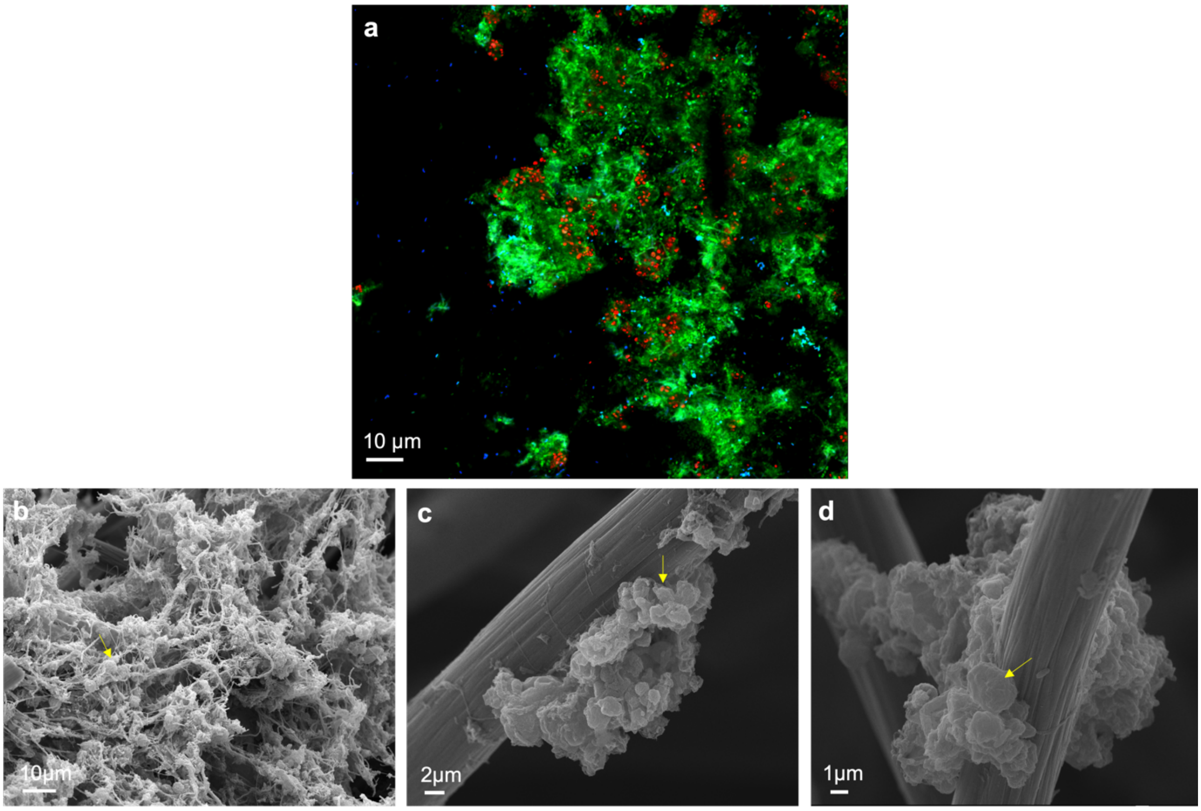

Supplementary Fig. 12 FISH and SEM micrographs of biofilms formed on the electrodes of BESs after 72 days of operation. (**a**) The FISH photomicrograph showing the *in situ* spatial distribution of '*Ca.* M. nitroreducens' (red), *Geobacter* (blue), and all bacteria (green). (**b**) SEM images showing biofilm colonization on the electrode (**c**) and direct association of '*Ca.* M. nitroreducens'-like cells with carbon fibers (**c, d**). Yellow arrows indicate the '*Ca*. M. nitroreducens'-like cells.

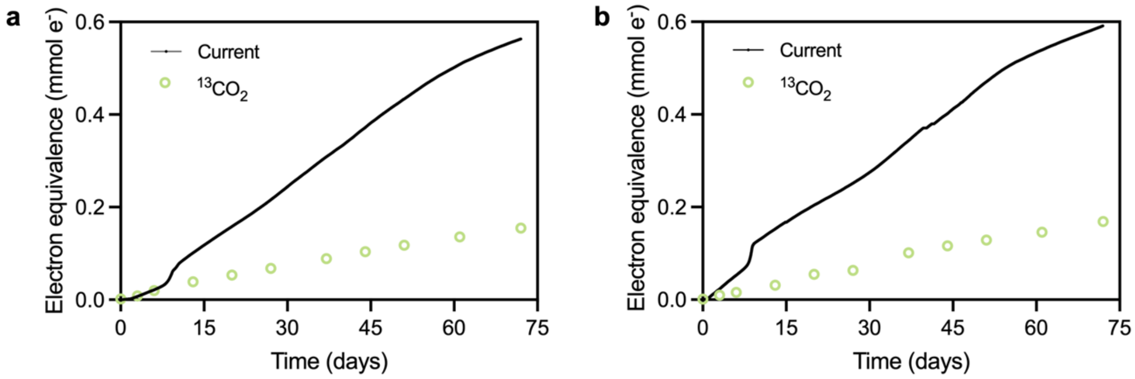

Supplementary Fig. 13 Current efficiency of electrode-dependent AOM in the BESs. Electron equivalence for the formation of measured $^{13}CO_2$ (from $^{13}CH_4$ oxidation) and electrons calculated from current generation in BES1 (**a**) and BES2 (**b**) are shown. Source data are provided as a Source Data file.

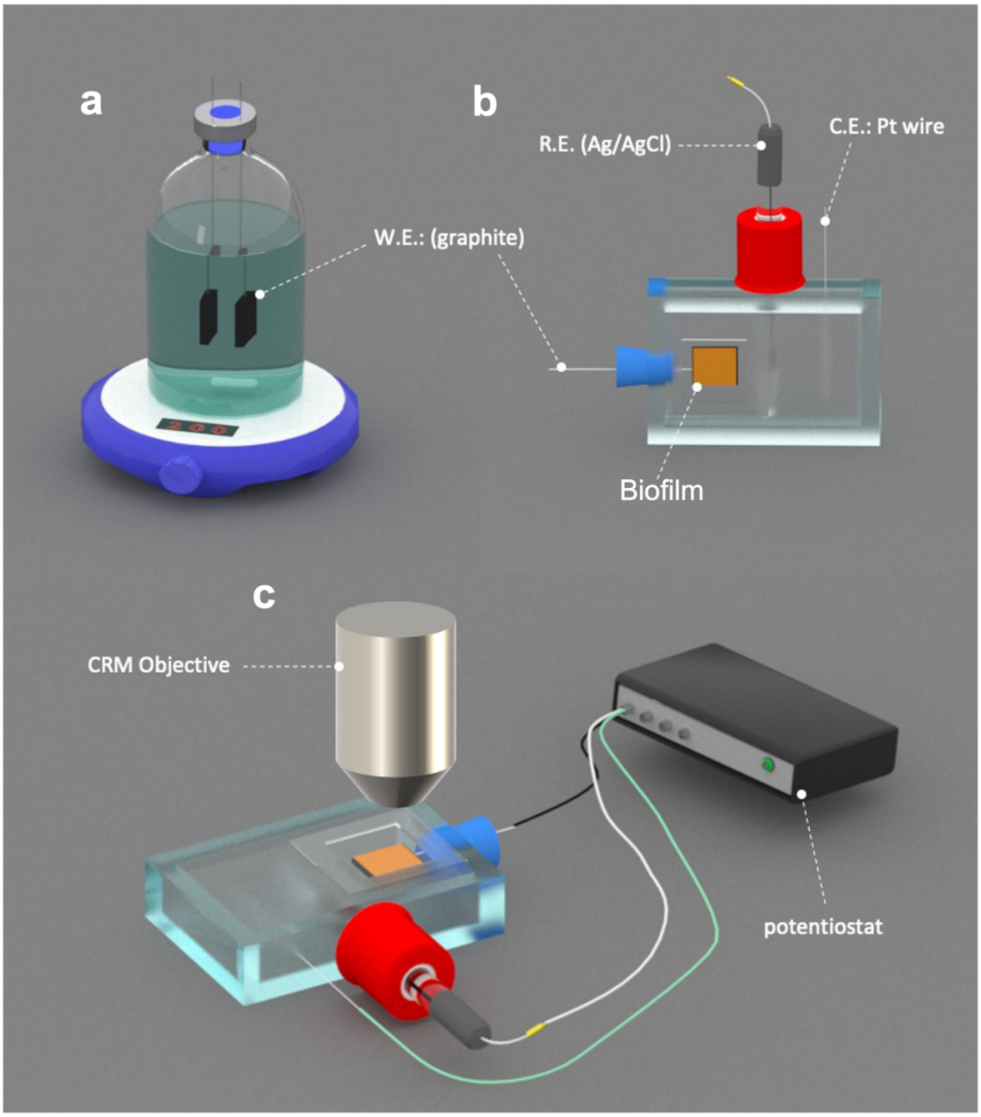

Supplementary Fig. 14 Schematic illustration of the progressive setup for Raman measurements. (**a**) Batch incubation setup for formation of '*Ca*. M. nitroreducens'-dominant biofilm on graphite electrodes. Graphite plates were polished using fine-grain sandpaper and then cleaned by sonication in acetone and RO water in sequence. A hole at 1 mm diameter was drilled on the side surface of a graphite plate, in which a titanium wire (diam. 0.5 mm, Sigma, USA) was inserted and fixed with conductive epoxy glue for electrical connection. Once completely dried, the exposed conductive glue was insulated by another waterproof epoxy glue. (**b**) The configuration of a spectroelectrochemical cell. Ports were opened on the side frame of the electrochemical cell to accommodate the working electrode (i.e., the electrode colonized with '*Ca*. M. nitroreducens' biofilm), the reference electrode and the counter electrode (consisting of a platinum wire). A window was opened on the side frame and a glass coverslip was glued on the frame to seal the window. The active internal volume of the cell was 15 mL. (**c**) Setup of the spectroelectrochemical cell for simultaneous electrochemical and resonance Raman measurements.

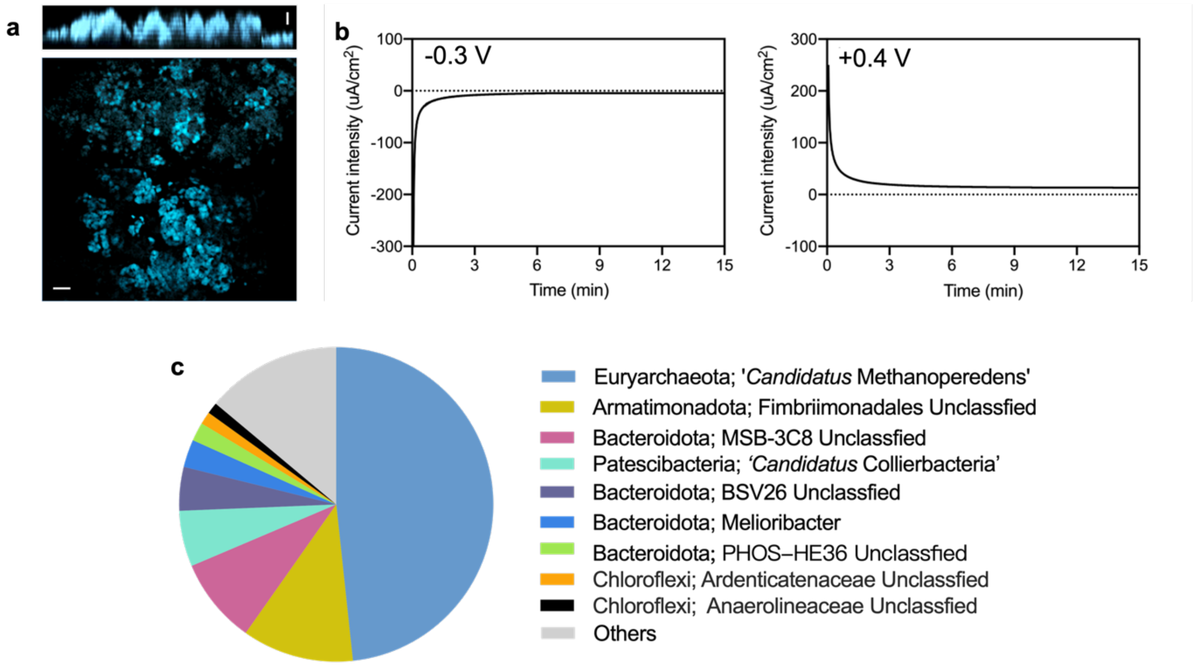

Supplementary Fig. 15 Biofilm characterization and current profiles of *in situ* spectroelectrochemical analysis with biofilm of '*Ca.* M. nitroreducens' enrichment culture. (**a**) A representative fluorescence microscopy image of '*Ca.* M. nitroreducens'-dominated biofilm formed on the graphite electrode after 128 days of incubation. Upper is a representative slice of the biofilm perpendicular to the biofilm surface. The mean biofilm thickness (n = 20) was 19.8 µm. Lower is a representative biofilm parallel to the electrode surface. Scale bar:10 µm. (**b**) A representative current profile for the electrochemical reduction (at -0.3 V *vs.* SHE) and oxidation (at +0.4 V *vs.* SHE) of biofilm of '*Ca.* M. nitroreducens' enrichment culture. Source data are provided as a Source Data file. (**c**) Microbial community structure of the biofilm after the Raman measurements. Taxa with an abundance of ⩾1% in at least one sample were presented. The biofilm from three duplicate electrodes was harvested for DNA extraction and 16S rRNA amplicon sequencing after Raman measurements were taken.
