# Supplementary material for: Multi-heme cytochrome-mediated extracellular electron transfer by the anaerobic methanotroph ‘Candidatus Methanoperedens nitroreducens’
Source: Nat Commun. 2023 Sep 30;14:6118. doi: 10.1038/s41467-023-41847-w (PMC10542353; doi:10.1038/s41467-023-41847-w)
Supplement: Supplementary file 3 — Description of additional supplementary files [file 41467_2023_41847_MOESM3_ESM.pdf]

Description of Additional Supplementary Files Document

Supplementary Data 1. Properties and relative metagenome and metatranscriptome abundances for all MAGs in this study

Supplementary Data 2. Differential gene expression for 'Ca. M. nitroreducens' with gene Annotations

Supplementary Data 3. Community TPM and annotations for Nitrate and Iron

Supplementary Data 4. Community TPM and annotations for Electrode

Supplementary Data 5. Community MHC TPM and annotations

Supplementary Data 6. Methanoperedens MHC details from pSORTb and SignalP
