# Supplementary material for: Multi-heme cytochrome-mediated extracellular electron transfer by the anaerobic methanotroph ‘Candidatus Methanoperedens nitroreducens’
Source: Nat Commun. 2023 Sep 30;14:6118. doi: 10.1038/s41467-023-41847-w (PMC10542353; doi:10.1038/s41467-023-41847-w)
Supplement: Supplementary file 10 — Reporting Summary [file 41467_2023_41847_MOESM10_ESM.pdf]

nature portfolio

Corresponding author(s): Shihu Hu

Last updated by author(s): Aug 29, 2023

Reporting Summary

Nature Portfolio wishes to improve the reproducibility of the work that we publish. This form provides structure for consistency and transparency in reporting. For further information on Nature Portfolio policies, see our Editorial Policies and the Editorial Policy Checklist.

Statistics

For all statistical analyses, confirm that the following items are present in the figure legend, table legend, main text, or Methods section.

n/a	Confirmed	
☐	☒	The exact sample size (*n*) for each experimental group/condition, given as a discrete number and unit of measurement
☐	☒	A statement on whether measurements were taken from distinct samples or whether the same sample was measured repeatedly
☐	☒	The statistical test(s) used AND whether they are one- or two-sided<br>*Only common tests should be described solely by name; describe more complex techniques in the Methods section.*
☒	☐	A description of all covariates tested
☒	☐	A description of any assumptions or corrections, such as tests of normality and adjustment for multiple comparisons
☐	☒	A full description of the statistical parameters including central tendency (e.g. means) or other basic estimates (e.g. regression coefficient) AND variation (e.g. standard deviation) or associated estimates of uncertainty (e.g. confidence intervals)
☐	☒	For null hypothesis testing, the test statistic (e.g. *F*, *t*, *r*) with confidence intervals, effect sizes, degrees of freedom and *P* value noted<br>*Give P values as exact values whenever suitable.*
☒	☐	For Bayesian analysis, information on the choice of priors and Markov chain Monte Carlo settings
☒	☐	For hierarchical and complex designs, identification of the appropriate level for tests and full reporting of outcomes
☒	☐	Estimates of effect sizes (e.g. Cohen's *d*, Pearson's *r*), indicating how they were calculated

Our web collection on statistics for biologists contains articles on many of the points above.

Software and code

Policy information about availability of computer code

Data collection	GC/MS data was collected with Chemstation software C.01.10 (Agilent, United States); Electrochemical analyses data was collected with the EC-Lab V11.30 of the VMP3 potentiostat (VMP3, Biologic Science Instrument, France) and CHI 1030 potentilstat (CH Instruments Inc, USA); The Raman resonance data was collected with the Project FOUR software (WITec GmbH, Ulm, Germany)
Data analysis	The following software were used for Data analysis: Chemstation software C.01.10, EC-Lab V11.30, GraphPad Prism 9.3.1 (471), Daime software 2.2; OriginPro 9.1, Cutadapt v4.2.0, Aviary v0.5.0 (https://github.com/rhysnewell/aviary), CoverM v0.6.1 (https://github.com/wwood/CoverM), DASTool, CheckM2, GTDB-Tk Classify function v2.1.0, SignalP v6.0, pSORTb v3.0.3, Kneaddata v0.10.0 (https://github.com/biobakery/kneaddata), Trimmomatic v0.39, StrandCheckR v1.15.0, Subread v2.0.3 package (http://subread.sourceforge.net/), DESeq2 v3.1592, pheatmap v1.0.12

For manuscripts utilizing custom algorithms or software that are central to the research but not yet described in published literature, software must be made available to editors and reviewers. We strongly encourage code deposition in a community repository (e.g. GitHub). See the Nature Portfolio guidelines for submitting code & software for further information.

Data

Policy information about availability of data

All manuscripts must include a data availability statement. This statement should provide the following information, where applicable:

- Accession codes, unique identifiers, or web links for publicly available datasets
- A description of any restrictions on data availability
- For clinical datasets or third party data, please ensure that the statement adheres to our policy

All raw metagenomic and metatranscriptomic reads generated in this study have been deposited in NCBI database with BioProject ID PRJNA1003560 (https://www.ncbi.nlm.nih.gov/bioproject/PRJNA1003560), BioSamples SAMN36897499 to SAMN36897508 (https://www.ncbi.nlm.nih.gov/biosample?LinkName=bioproject_biosample&from_uid=1003560), and Sequence Read Archive IDs SRR25580091 to SRR25580106 (https://www.ncbi.nlm.nih.gov/sra?LinkName=bioproject_sra_all&from_uid=1003560). The differential gene expression analysis are summarized in Datasets in the Supplementary Data 2.

Research involving human participants, their data, or biological material

Policy information about studies with human participants or human data. See also policy information about sex, gender (identity/presentation), and sexual orientation and race, ethnicity and racism.

Reporting on sex and gender	N/A
Reporting on race, ethnicity, or other socially relevant groupings	N/A
Population characteristics	N/A
Recruitment	N/A
Ethics oversight	N/A

Note that full information on the approval of the study protocol must also be provided in the manuscript.

Field-specific reporting

Please select the one below that is the best fit for your research. If you are not sure, read the appropriate sections before making your selection.

☒ Life sciences ☐ Behavioural & social sciences ☐ Ecological, evolutionary & environmental sciences

For a reference copy of the document with all sections, see nature.com/documents/nr-reporting-summary-flat.pdf

Life sciences study design

All studies must disclose on these points even when the disclosure is negative.

Sample size	The sample size was selected by the objective of the reproducibility of experiments and the statistics analyses of gene expression across the nitrate, iron, and electrode conditions. All sample size performed in each experiment is indicated in the Method section. In general, sample size of n=3 was chosen for the chemical analyses, metagenomic and metatranscriptomic analyses, fluorescence assays and TEM visualization of iron- and nitrate-reducing experiments. Sample size of n=6 was used for the silver-reducing experiments, and n=4 independent sample was used for Raman spectroscopic measurements. For the long-term bioelectrochemical experiment, n=2 independent biological replicates were performed and n=3 replicate samples (each with sample size of n=5 from different electrodes) was used for metagenomic and metatranscriptomic analyses.
Data exclusions	No data were excluded from the analysis.
Replication	All experiments were independently repeated. Both the iron reduction and nitrate reduction batches were conducted with n=3 independent cultures for performance and chemical analyses. Metagenomics and metatranscriptomics analyses for iron and nitrate reduction batches were conducted with n=3 independent cultures. The representative fluorescent images for iron-reducing incubation were selected from n=3 independent experiments, and similar results were repeated from independent experiments. The congruency of iron-reducing cells and 'Ca. M. nitroreducens' were calculated from n=20 fields of view through samples from n=3 independent experiments. The representative TEM images for cytochrome staining were selected from n=3 independent experiments and similar results were repeated from independent experiments. The silver-reducing experiment was conducted with n=6 independent cultures, and similar TEM images of silver nanopartice deposition were repeated through 6 independent experiments. Representative current density profiles and cyclic voltammetry curves in electrochemical analyses were selected from n=3 independent tests. The Raman measurements were performed with n=4 independent electrode biofilm samples. The long-term bioelectrochemical systems were operated with n=2 independent experiments, which showed repeated performance. Metagenomics and metatranscriptomics analyses for bioelectrochemical systems were conducted with n=3 independent replicative samples, each with n=5 biofilm samples.<br>All attempts at replication were successful.

Randomization	The experiments were not randomized, since all experiments (iron- and nitrate-reducing experiments, operation of long-term bioelectrochemical systems, electrochemical characterization and Raman measurement), image visualization (fluorescence imaging, TEM, SEM) and analyses (meta-omics data analyses) in this study were based on specific enrichment culture maintained in the lab with replicates.
Blinding	No blinding was performed in this study. All experiments were conducted with a unique anaerobic enrichment culture maintained in the bioreactor, which made blinding such studies not applicable.

Reporting for specific materials, systems and methods

We require information from authors about some types of materials, experimental systems and methods used in many studies. Here, indicate whether each material, system or method listed is relevant to your study. If you are not sure if a list item applies to your research, read the appropriate section before selecting a response.

Materials & experimental systems

n/a	Involved in the study
☒	☐ Antibodies
☒	☐ Eukaryotic cell lines
☒	☐ Palaeontology and archaeology
☒	☐ Animals and other organisms
☒	☐ Clinical data
☒	☐ Dual use research of concern
☒	☐ Plants

Methods

n/a	Involved in the study
☒	☐ ChIP-seq
☒	☐ Flow cytometry
☒	☐ MRI-based neuroimaging
